# Dr. Benjamin F. Sherman

**Published:** 1897-08

**Authors:** 


Dr. Benjamin F. Sherman, of Ogdensburg, died at his home in that city May 30, 1897, aged 80 years. He was the most prominent physician in his section of the state, and by his amiability of temper, professional accomplishment, dignity of character and courteous manners, contributed largely to the *esprit de corps* of the medical profession of Northern New York.

Dr. Sherman was elected president of the Medical Society of the State of New York in 1884, in the midst of the now almost forgotten code controversy, and his espousal of the new code contributed not a little to its successful adoption and solid maintenance. His opinion was often sought as a consultant at great distances, and he retained his association and popularity with young people even to his end. He was the dean of the Medical Society of the State of New York and he had seldom been absent from its meetings during the period of his membership—nearly a third of a century. His tall, commanding presence, his dignified bearing and his urbanity of manner will be greatly missed in the future councils of the society.

